# DSMZCellDive: Diving into high-throughput cell line data

**DOI:** 10.12688/f1000research.111175.2

**Published:** 2022-07-20

**Authors:** Julia Koblitz, Wilhelm G. Dirks, Sonja Eberth, Stefan Nagel, Laura Steenpass, Claudia Pommerenke

**Affiliations:** 1 Leibniz Institute DSMZ - German Collection of Microorganisms and Cell Cultures, Braunschweig, 38124, Germany

**Keywords:** DSMZ, human and animal cell lines, RNA-seq, STR, HLA, omics data, LL-100, leukemia, lymphoma, homeobox

## Abstract

Human and animal cell lines serve as model systems in a wide range of life sciences such as cancer and infection research or drug screening. Reproducible data are highly dependent on authenticated, contaminant-free cell lines, no better delivered than by the official and certified biorepositories. Offering a web portal to high-throughput information on these model systems will facilitate working with and comparing to these references by data otherwise dispersed at different sources.

We here provide DSMZCellDive to access a comprehensive data source on human and animal cell lines, freely available at celldive.dsmz.de. A wide variety of data sources are generated such as RNA-seq transcriptome data and STR (short tandem repeats) profiles. Several starting points ease entering the database via browsing, searching or visualising. This web tool is designed for further expansion on meta and high-throughput data to be generated in future. Explicated examples for the power of this novel tool include analysis of B-cell differentiation markers, homeo-oncogene expression, and measurement of genomic loss of heterozygosities by an enlarged STR panel of 17 loci.

Sharing the data on cell lines by the biorepository itself will be of benefit to the scientific community  since it (1) supports the selection of appropriate model cell lines, (2) ensures reliability, (3) avoids misleading data, (4) saves on additional experimentals, and (5) serves as reference for genomic and gene expression data.

## Introduction

For more than 70 years, cell lines have become indispensable for life sciences, especially for biomedical research. Human and animal cell lines represent cost-effective model systems of almost unlimited availability. Furthermore, they are relatively easy to manipulate. The vast majority of cell lines are derived from spontaneously immortalised tumour cells carrying specific characteristics. They have become essential models not only for cancer research such as investigating mechanisms of tumorigenesis, targets for therapy, or drug efficacies but also for other areas of biological sciences. Cell lines are made available to the scientific community by cell lines banks, who also control and guarantee for cell line authenticity. In general both, diagnosis and clinical parameters of the donor as well as molecular characteristics of the cell line itself, are required for the selection of an appropriate model cell line for a specific research question.

Usually, the required information about cell lines has to be gathered from different sources, often including the study of literature. In addition to the information provided in the cell line data sheets of the biorepositories themselves, several online databases independent of the biorepositories can be consulted. The largest available online platform listing key information and references from continuous cell lines is Cellosaurus ^1^. However, so far the mentioned sources contain only limited information about genetic or transcriptional aberrations characterising a cell line. Today, molecular characteristics obtained from high-throughput sequencing data from cell lines become more and more relevant for the selection of appropriate models. In this context, the cell lines project database of COSMIC (Catalogue of Somatic Mutations in Cancer) provides mutation profiles and copy number variations of over 1,000 cancer cell lines ^2^. Further publicly accessible datasets for download are multiple high-throughput sequencing data from the CCLE (Cancer Cell Lines Encyclopedia) panel *via* the DepMap Portal which also offers interactive data visualisation ^3^. In order to support the search for appropriate cell line models Jeong *et al.* developed the online database GEMiCCL which enables the comparison of genomic, transcriptomic and copy number data generated in different projects, including CCLE, COSMIC and the NCI-60 cell lines panel ^4^. Importantly, the comparison of the data from different sources points out that *e.g.* mutation data can strongly vary for a cell line depending on the data source. This observation is not surprising, as selective culture conditions can foster evolution of cell lines, impacting the genetic and transcriptional diversity of the same cell line between two laboratories ^5^. Thus, the major drawbacks during the selection process for an appropriate model cell line are either that the molecular data cannot easily be traced back to the source of the cell line and culture conditions used for the generation of the data, and that bioinformatics skills are required to re-analyse publicly available omics data after downloading.

So far, most molecular data including high-throughput sequencing data are available from different sources but not from the cell line repository itself. This fact ultimately confronts the selection of cell lines with the question from which resource the cell line with the appropriate molecular characteristics is actually available. To overcome this limitation we developed DSMZCellDive, a novel web portal that offers access to evaluated RNA-seq data and STR profiles generated from material of human and animal cell lines that are provided to the scientific community via the DSMZ catalogue.

## Methods

### Implementation

All data were provided by internal sources at the DSMZ, extracted from various formats using tailored scripts, and finally transferred into an SQL database. The data were integrated by using cell culture identifiers, such as the name of the cell line and DSMZ ACC-No. The SQL database is based on MariaDB Version 10.3. PHP 7 is used to generate the web pages and to query an internal SQL database. The JavaScript libraries jQuery and Plotly.js are used to do asynchronous server requests and draw charts, respectively. Heat maps are created using the R library heatmaply. The PHP library Parsedown is used to display the COI barcoding report.

The importance of machine-readability is increasing steadily. So far, web search engines use a standardised vocabulary ( schema.org) to do basic queries. The Bioschemas initiative ( bioschemas.org) aims to extend these vocabularies for life sciences. We have integrated Bioschemas profiles and added a machine-readable markup for all cell lines. The Bioschemas representations enhance interoperability and standardisation of DSMZCellDive.

### Operation

DSMZCellDive can be accessed at celldive.dsmz.de with every modern browser and is free of charge.

## Results

### Data sources

DSMZCellDive is designed to provide a data portal to high-throughput and meta data of cell lines hosting diverse data sources. Gene expression data beside HLA (human leukocyte antigen) information, STR (short tandem repeat) profiles, and COI (cytochrome c oxidase I) DNA barcodes are bundled at start as listed in Table 1 and there is more to come. In the following current data sources are described briefly.

**Table 1.** Current data in DSMZCellDive. *100 leukemia and lymphoma cell lines + NC-NC, a B lymphoblastoid cell line.

Data source	type	# cell lines	Reference
LL-100	RNA-seq	101 *	6
LL-100	HLA	101 *	6
STR	STR profiles	4565	10
COI	COI barcodes	197	-

The vast majority of data entries in DSMZCellDive is composed of RNA-seq data. We started with our published data on 100 human leukemia and lymphoma cell lines (LL-100 panel) ^6^. In contrast to the cited paper, the non-malignant cell line NC-NC, a B lymphoblastoid cell line, is included and RNA-seq data were quantified *via* Salmon ^7^ and normalised *via* DESeq2 ^8^ in order to keep pace with state-of-the-art data analysis.

HLA genes encode proteins in the major histocompatibility complex (MHC) which play a central role in discriminating self and non-self ^9^. Although the HLA gene cluster on chromosome 6 is highly polymorphic, it is not suitable for cell line authentication due to a low exclusion rate and instability of gene expression. Furthermore, HLA typing is important for cancer research since determination of tissue compatibility by tumour neoantigen binding to HLA surface proteins and rejection of specific HLA alleles play a role. Here, HLA typing was determined on LL-100 RNA-seq data *via* arcasHLA, an alignment-based tool ^9^.

The applied genotyping system (Promega Powerplex 18D) uses STR microsatellite repeats which are located at 17 specific genomic loci that are highly polymorphic in human populations including gender determination *via* Amelogenin. STR typing is serving as a reference technique for identity control of human cell lines at biological resource centers and available as global standard (ANSI/ATCC ASN-0002-2021 (2021). Authentication Of Human Cell Lines: Standardization Of Short Tandem Repeat (STR) Profiling. ANSI eStandards Store). Data sources were kindly provided by ATCC, JCRB, and amended by DSMZ ^10^.

DNA barcoding for animal samples is frequently based on the Cytochrome c oxidase subunit 1 (COI or COX1) DNA sequence known to exert specific differences between species - prerequisite for species identification. DSMZCellDive harbours COI barcodes for all animal cell lines available at the DSMZ.

### Data structure

**Figure 1.**
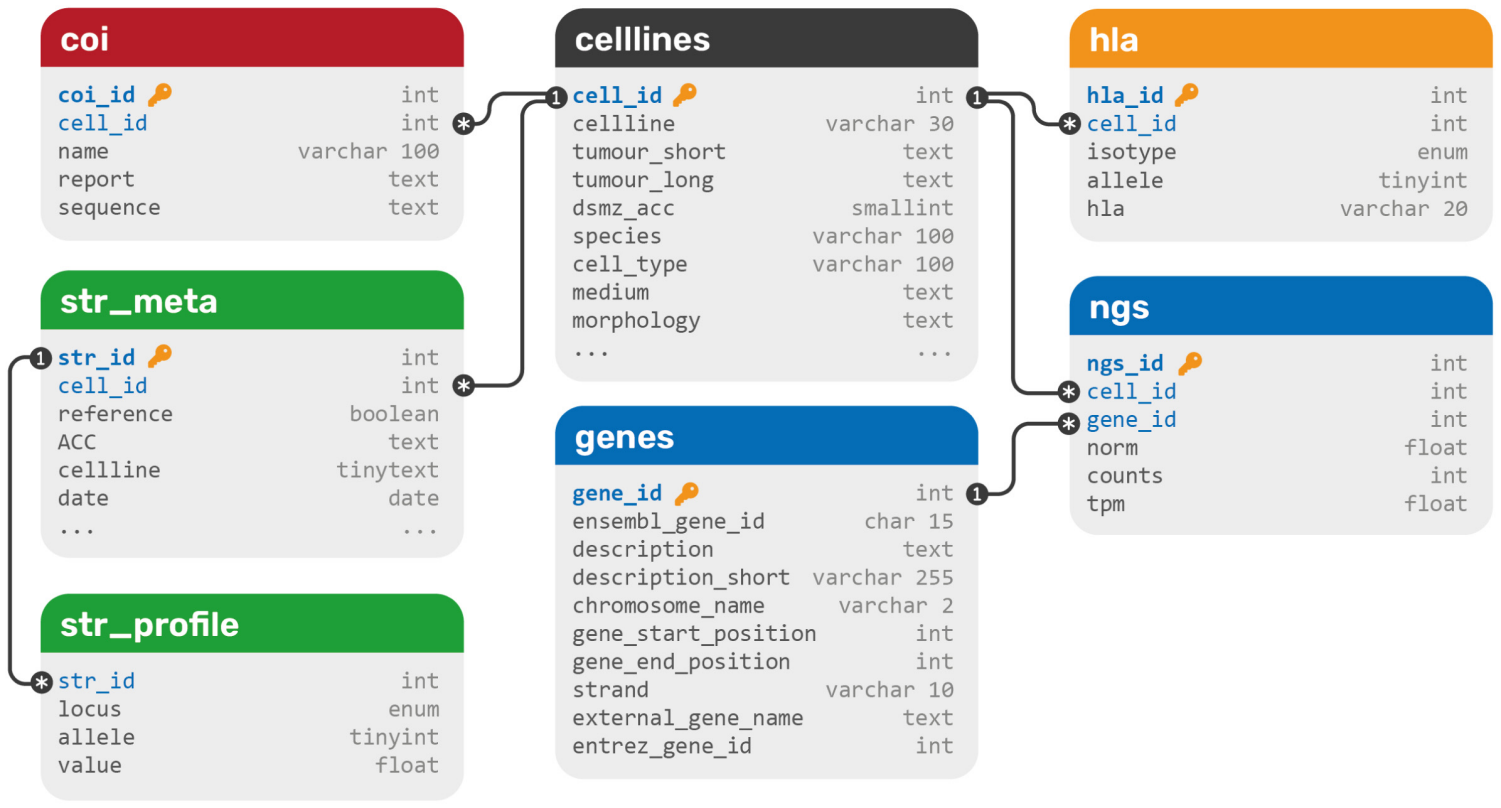
The database structure of DSMZCellDive. Primary keys are blue and marked with a key symbol, foreign keys are blue. Each color represents a different data type: blue for RNA-seq data, green for STR profiles, yellow for HLA typing data, and red for COI barcoding data. All data sets are integrated via a central cell line table (black).

All data were extracted from various data formats and integrated into a relational SQL database. The database structure contains seven tables in total and can be extended with more data types easily ( Figure 1). All data types that belong to DSMZ cell lines are connected to the celllines table *via* one-to-many connections to its primary key cell_id. Since one cell line can have multiple STR profiles, a meta table is used to connect profiles to cell lines. This should not be confused with the fact that one cell line may carry multiple STR alleles per STR loci, a phenomenon called microsatellite instability (MSI). The whole COI barcoding DNA sequence as well as the report is saved as markdown in a single text field as currently no search or comparison operations are planned on these data, since species-specificity only not genetic individuality is resolved by this technique.

### Website data portal

The DSMZCellDive website was designed to be easily extensible by more data types. For this reason, the web layout contains a sidebar instead of a classical navbar, as it allows more content. The header contains breadcrumbs to meet the requirements of the hierarchical structure of the page. The starting page gives an overview on all available data types and provides links to all tables and tools. Each data source has its own overview page, describing the data, available literature and providing further links.

Each RNA-seq data set has its own entry page and supports visualisation as bar chart or heat map. An interactive web interface enables the user to enter genes (up to five for bar charts and up to 20 for heat maps), select tumour entities or cell lines directly, and choose whether normalised data, raw counts, or TPM (transcripts per kilobase million) values should be used. Currently, only RNA-seq data from the LL-100 panel are available; however, it is planned to add more data sets in the near future. For this reason, an integrated view on all RNA-seq data from the different projects was added early on. Since data are normalised within each project, normalised values between projects should not be compared, only TPM data and bar charts are available here.

The STR data sets come with a browser and a search tool. While the browser allows access to unique comprehensive STR data sets of DSMZ and other major cell lines banks, the search tool allows users to compare own STR profiles for similarity matching with regard to authenticity. As a result of the search closest matches according to the following equation are displayed and the ratio to the reference is given in percent distance as described previously ^11^.

$$\frac{2\cdot {\text{A}}_{shared}}{{\text{A}}_{query}+{\text{A}}_{ref}}\;(1)$$

The result page offers further information on the cell lines and is cross-linked to the respective cell line detail page, which also enables access to the cell line data sheet of the DSMZ catalogue.

All data are integrated on cell line level. Each of the currently almost 900 cell lines has its own detail page, including meta data on the cell line, *i.e.* the species, cell type, morphology, and culture media. Depending on whether it is a human or animal cell line, STR profiles or COI DNA barcode reports are displayed, respectively. If the cell line belongs to any RNA-seq project, a histogram of all genes is shown, as well as HLA typing data.

## Use cases

### Analysis of B-cell differentiation marker genes

**Figure 2.**
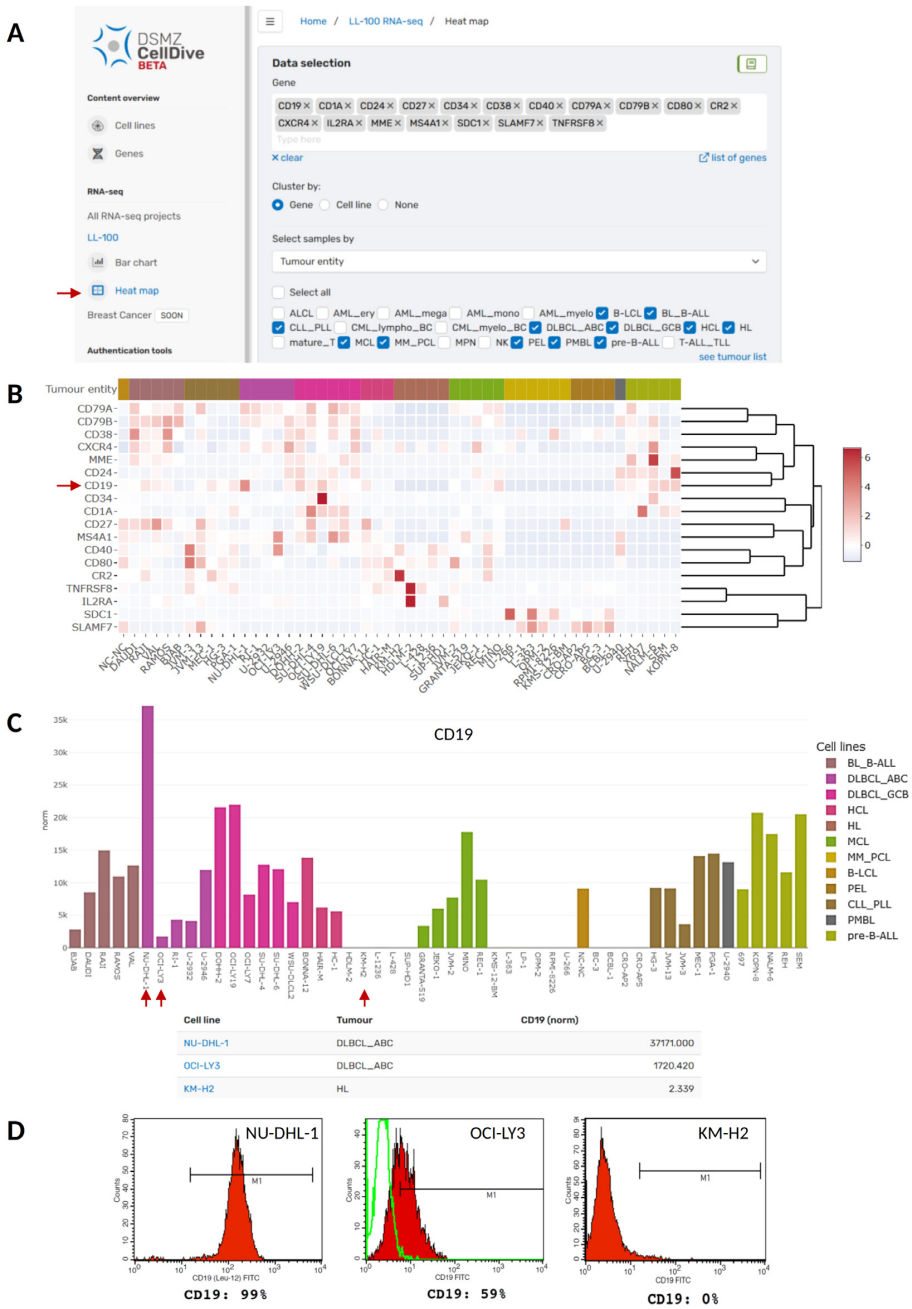
Analysis of B-cell marker genes in B-cell derived cancer cell lines of the LL-100 panel using DSMZCellDive. ( **A**) For customised analysis of RNA-seq data of the LL-100 project via the new web tool 18 genes and 12 tumour entities were selected to visualise expression pattern in a heat map (red arrow). ( **B**) The resulting heat map depicts normalised expression of the genes in rows within the selected cell lines depicted in columns. Red arrow: *CD19*. ( **C**) Alternatively, expression of single genes can be visualised in a bar chart. As an example normalised expression of *CD19* (indicated by an arrow in **B**) is shown in the same tumour entities selected in **A** and **B**. Additionally, a table listing the values for normalised *CD19* expression per cell line appears underneath the bar chart. ( **D**) Protein expression of CD19 in three of the cell lines depicted in **C** (red arrows). Flow cytometry data were taken from the corresponding cell line data sheets of the DSMZ catalogue to which users are guided by clicking on the blue cell line names in the table depicted in **C**.

Our new web tool can be applied to analyse and visualise the expression of B-cell development and differentiation markers in B-cell derived cancer cell lines ( Figure 2). B-cells originate from multipotent hematopoietic stem cells in the bone marrow and undergo a series of antigen-independent and antigen–dependent differentiation and maturation steps until they finally become memory or plasma B-cells that secrete antibodies. These steps are associated with the expression of well-studied marker genes, many of them encode for cell surface molecules commonly used for immunophenotyping. Importantly, B-cell derived cancer cells still mirror the differentiation and maturation phase of their normal B-cell counterpart, which is also reflected in the expression profile of B-cell marker genes and is crucial for the diagnosis of B-cell neoplasms ^12, 13^. Figure 2B depicts a heat map illustrating the expression of a customised selection of 18 B-cell differentiation marker genes ( *CD1A, CD19, CD24, CD27, CD34, CD38, CD40, CD79A, CD79B, CD80, CR2, CXCR4, IL2RA, MME, MS4A1, SDC1, SLAMF7*, and *TNFRSF8*) across the B-cell derived cell lines included in the LL-100 panel. Intriguingly, specific B-cell markers get lost in the tumour cells. For example, although CD19 is usually expressed in all B-cells from pre-B-cells until the terminal differentiation to plasma cells ^14^, its expression is typically absent in Hodgkin lymphoma (HL), plasma cell leukemia (PCL), primary effusion lymphoma (PEL) and in a fraction of Diffuse large B-cell lymphoma (DLBCL) ^15, 16^. Accordingly, *CD19* loss is also seen in cell lines representing HL, PCL, and PEL ( Figure 2C). The DLBCL cell line OCI-LY3 is an example for weak CD19 expression on mRNA and protein level compared to DLBCL cell line NU-DHL-1 harboring the highest CD19 expression across all analyzed cell lines ( Figure 2C and 2D). Thus, DSMZCellDive can assist the selection of model cell lines *e.g.* with varying levels of *CD19*.

### NKL homeobox gene analysis

Homeobox genes encode transcription factors sharing a special helix-turn-helix 3D-structure which mediates interaction with DNA, cofactors and chromatin. This homeodomain is formed by 60 amino acid residues and represents a platform performing gene regulation. Homeobox genes control fundamental processes in development and differentiation during embryogenesis and in the adulthood ^17^. Therefore, deregulation of their activity is a common theme in cancer including hematopoietic malignancies.

According to their conserved homeobox sequences, these genes are arranged in eleven classes and several subclasses ^18^. The NKL subclass (NK-like) belongs to the ANTP class (according to the *Drosophila antennapedia* gene) and consists of 48 members in humans. Their physiological expression pattern in the hematopoietic compartment has been termed “NKL-code“ comprising eleven genes ^19^. This code is a useful tool to evaluate deregulated NKL homeobox genes in myeloid and lymphoid leukemia/lymphoma patients. To date, 24 aberrantly activated NKL homeobox genes are described in T-cell acute lymphoblastic leukemia (T-ALL), representing the strongest group of oncogenes in this malignancy ^20^. TLX1 (formerly HOX11) and TLX3 (HOX11L2) are the most frequently deregulated NKL homeobox genes in T-ALL while NKX2-5 is only rarely expressed ^19^.

**Figure 3.**
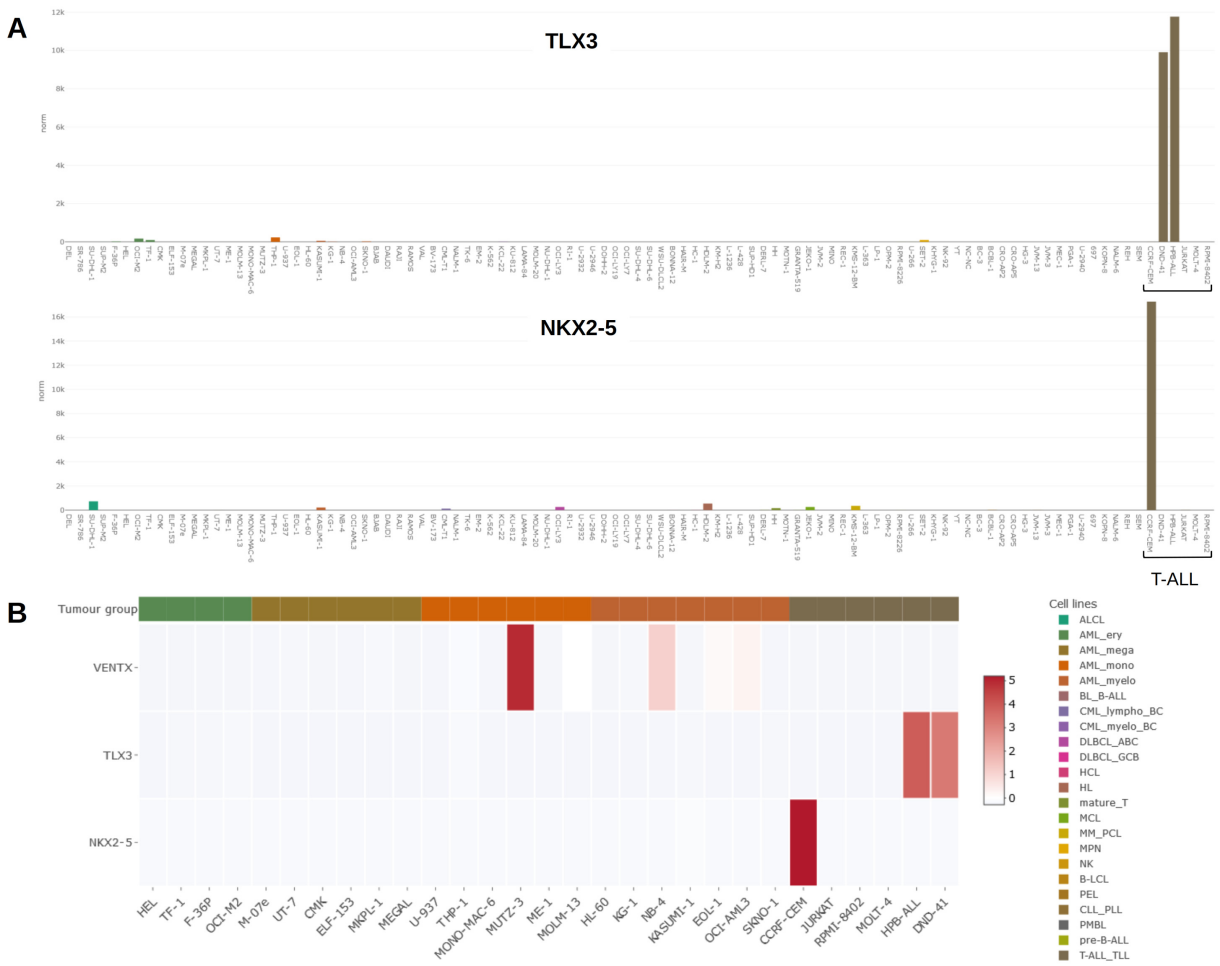
( **A**) Bar plots showing activities for NKL homeobox genes *TLX3* (above) and *NKX2-5* (below) in the LL-100 panel. Both genes are exclusively expressed in T-ALL cell lines DND-41, HPB-ALL ( *TLX3*) and CCRF-CEM ( *NKX2-5*). T-ALL cell lines are indicated by a bracket. ( **B**) Heat map showing activities for NKL homeobox genes *VENTX, TLX3* and *NKX2-5* in AML and T-ALL cell lines (left). *VENTX* is prominently expressed in myelomonocytic cell line MUTZ-3, representing a model for conventional dendritic cells. A color-code assigns tumour entities to the cell lines as indicated in **A** and **B** (right).

Cell lines are useful models to investigate regulation and function of oncogenes including homeobox genes. To identify a leukemia/lymphoma cell line expressing a particular homeo-oncogene, our published LL-100 dataset and the here presented online tool DSMZCellDive may assist to find a suitable cell line model ^6^. Figure 3A illustrates generated results, showing gene expression data for *TLX3* and *NKX2-5* as barplots. Both genes are expressed in particular T-ALL cell lines while silenced in cell lines derived from other leukemia/lymphoma entities ^21, 22^. NKL homeobox gene VENTX is physiologically expressed in lymphoid T-cell progenitors and myeloid conventional dendritic cells (cDC) ^23^. MUTZ-3 is an AML (acute myeloid leukemia) cell line derived from a cDC progenitor. This cell line expresses VENTX and represents a useful model to investigate differentiation of dendritic cells and their derived malignancies ^23^. Figure 3B shows NKL homeobox gene activities for *VENTX, TLX3* and *NKX2-5* in selected cell lines as heat map, demonstrating strikingly high *VENTX* expression in MUTZ-3. Thus, DSMZcellDive is a useful tool to identify and illustrate normal and aberrant (homeobox) gene expression in leukemia/lymphoma cell lines.

### Importance of drifted or lost STR alleles in cell lines

Although *in vitro* evolution of tumour cell lines is well known ^5^, the underlying genomic alterations often remain obscure. Despite elaborate quality control via STR genotyping of lot charges, crucial genetic changes of a tumour model may remain hidden to the applying scientist.

**Figure 4.**
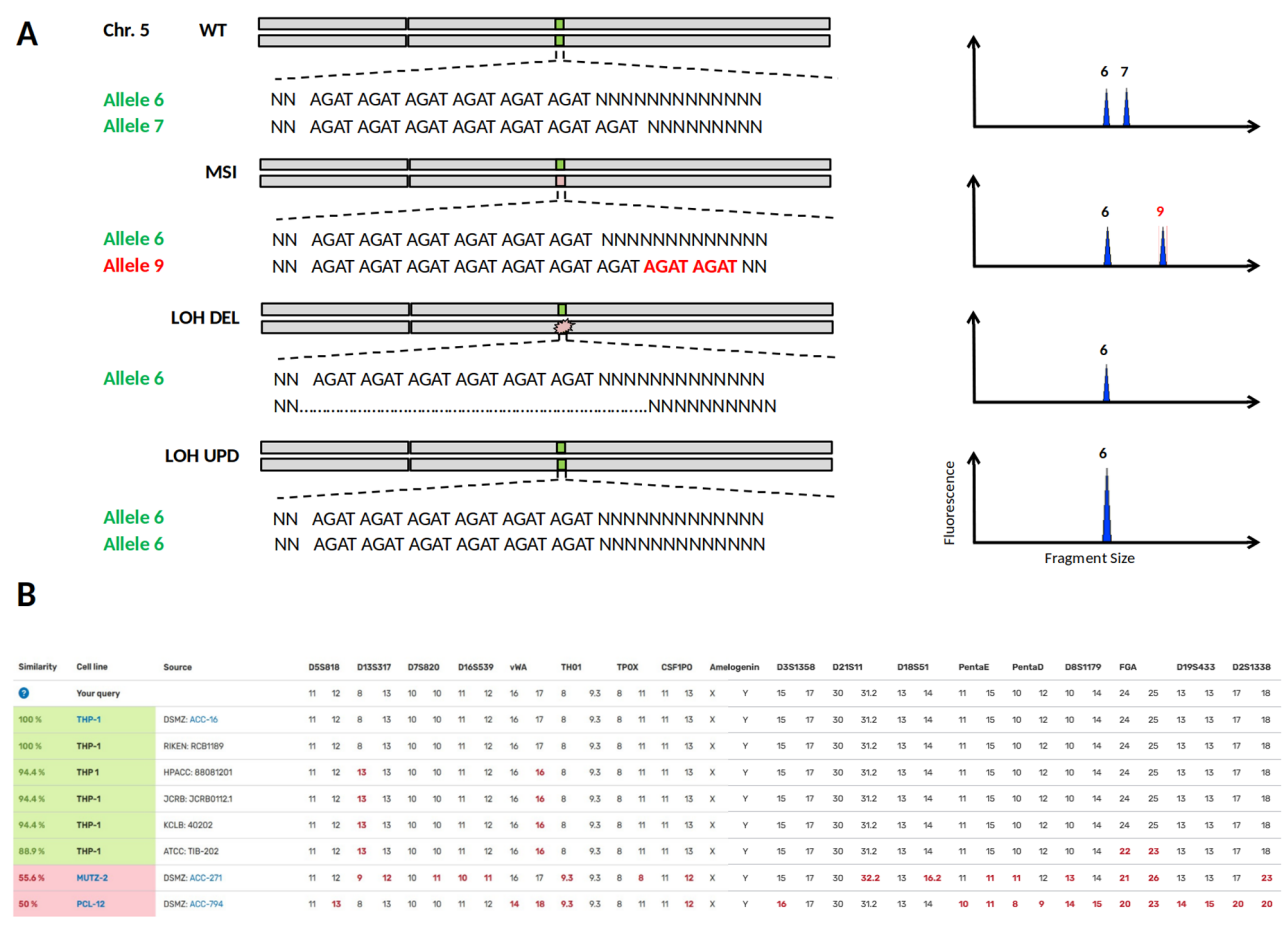
Loss of heterozygosity (LOH) or Microsatellite Instability (MSI) in STR profiles of cell lines. ( **A**) In the upper half the heterozygous wild type status of STR locus D5S818 on chromosome 5 is shown, corresponding STR electropherograms are depicted on the right. MSI is caused by a replication error during DNA synthesis and may result in a new allele 9 by allelic drift. LOH can occur by a deletion mutation (LOH DEL) or by recombination between paternal chromosomes known as uniparental disomy (UPD). In both LOH cases, genomic DNA is lost and may lead to undesired losses of particular physiological properties at worst. ( **B**) Searching for the reference STR profile of the cell line THP-1 via DSMZCellDive delivers similarity scores to other THP-1 profiles and deviating/missing alleles are shown as red numbers. Cell lines highlighted in green indicate authenticity without doubt, while hits below 60% matching similarity marked in pink are unrelated and thought to be definitely genetically different.

The importance of MSI and LOH (loss of heterozygosity) can be demonstrated in one of the most commonly used models for AML research, the cytokine-dependent cell line THP-1. A recent publication by Noronha *et al.* describes a large genetic divergence between THP-1 cells from a European and a US biorepository ^24^. Although globally standardised STR genotyping in biorepositories is primarily used to prevent the spread of misidentified cell lines, STR typing can distinguish subclones from parental cell lines when minor changes in the STR profile have occurred due to MSI or LOH ( Figure 4A). Specifically, it is LOH changes that may qualitatively reveal the loss of a heterozygous chromosomal region but do not allow quantitative conclusions to be drawn. For the divergent THP-1 cell lines, the STR profiles search *via* DSMZCellDive using 17 STR loci yield similarities of 94.4% and 88.9%, respectively ( Figure 4B), indicating a close genetic relationship between THP-1 cell lines of different repositories. Despite these high similarities, critical genetic targets of MLL (mixed lineage leukemia) differed substantially between cell bank-specific THP-1 cells ^24^. Thus, not only is cross-contamination of cell lines a serious problem for the reproducibility of scientific data, but also silent LOH events within a scientific tumour model.

Thus, DSMZCellDive enables scientists to verify the authenticity of cell lines by providing an extended STR-17 panel, which measures the degree of correspondence between the original cell line and already slightly modified cell lines. Since all commonly measured STR loci for authentication are combined in the STR-17 panel, this is independent of the STR typing kits used. By presenting the diploid STR datasets in two columns, LOH and UPD events can be deduced, which in turn immediately shows users how often and at what point their own STR data has deviated from the STR reference profile. The described tools will increase reliability of *in vitro* data towards trusted scientific conclusions.

## Conclusion

With the interactive web portal DSMZCellDive at hand, access to cell lines omics and meta data is bundled at one site. DSMZCellDive presents its own RNA-seq data exclusively from cell lines of the DSMZ. The novel web portal allows researchers to browse and visualize authentic NGS data generated according to high quality standards *e.g.* to support the selection of appropriate model cell lines, while cell lines are acquired. Furthermore, its concept allows implementation of future data readily and, more importantly, provides reliable data of cell lines available at DSMZ, which can serve as reference data for industry and science.

## Data and software availability

### Underlying data

All data underlying the results are available as part of DSMZCellDive and no additional source data are required.

### Software availability

Website: https://celldive.dsmz.de

Source code at github to download and process: 

Website: https://github.com/JKoblitz/DSMZCellDive

 RNA-seq: https://github.com/claupomm/RNA-seq_ll100

HLA analysis: https://github.com/claupomm/HLA-analysis

Zenodo for archived source code at time of publication:

Website: https://doi.org/10.5281/zenodo.6404422

RNA-seq pipeline: https://doi.org/10.5281/zenodo.6401600

HLA analysis: https://doi.org/10.5281/zenodo.6401594

License: MIT License
