# Supplementary material for: DSMZCellDive: Diving into high-throughput cell line data
Source: F1000Res. 2022 Jul 20;11:420. Originally published 2022 Apr 13. [Version 2] doi: 10.12688/f1000research.111175.2 (PMC9334839; doi:10.12688/f1000research.111175.2)
Supplement: Peer review history

# Peer review: Reviewer response for version 2

**DOI:** 10.5256/f1000research.136217.r144763

**Published:** 2022-07-28

**Authors:** Arihiro Kohara

**Affiliations:** 1 Laboratory of Cell Cultures, National Institutes of Biomedical Innovation, Health and Nutrition, Osaka, Japan

I confirmed that I had read this submission and found that the author had properly modified the manuscript.

Are the conclusions about the tool and its performance adequately supported by the findings presented in the article?

Yes

Is the rationale for developing the new software tool clearly explained?

Yes

Is the description of the software tool technically sound?

Yes

Are sufficient details of the code, methods and analysis (if applicable) provided to allow replication of the software development and its use by others?

Yes

Is sufficient information provided to allow interpretation of the expected output datasets and any results generated using the tool?

Yes

Reviewer Expertise:

cell biology, genetic mutation

I confirm that I have read this submission and believe that I have an appropriate level of expertise to confirm that it is of an acceptable scientific standard.

# Peer review: Reviewer response for version 1

**DOI:** 10.5256/f1000research.122868.r135173

**Published:** 2022-07-06

**Authors:** Arihiro Kohara

**Affiliations:** 1 Laboratory of Cell Cultures, National Institutes of Biomedical Innovation, Health and Nutrition, Osaka, Japan

In this manuscript, the authors are concerned with the creation of a new web portal that creates a database of cell metadata and characteristic analysis information in cell banks and biorepositories, and will be able to provide useful information for researchers using cell lines. This manuscript can be highly evaluated as an initiative. We also hope that information such as RNA-seq will be expanded in the future, and that fusion gene search based on sequence information and data sharing with other banks will also be expected.

 However, I think it will be a better manuscript if you reconsider the following points, so please consider modifying it.

**Major**

- In the RNA-seq heat map analysis, the clustering results performed based on the similarity of the expression profile of each gene are displayed, but the clustering result based on the similarity of the expression profile of each cell line should also be displayed.

**Minor**

- The legend in Fig. 4B is not appropriate. There are no cell lines highlighted in yellow, while there is no indication of the color of cells highlighted in red.
- Although the data in Fig. 4B shows only 9 loci data as an example of LOH, it is considered that 17 loci data is collected in this database, so please describe the verification using all the data.
- Also, why not explain the example of MSI-H using MOLT-4 etc.?

Are the conclusions about the tool and its performance adequately supported by the findings presented in the article?

Yes

Is the rationale for developing the new software tool clearly explained?

Yes

Is the description of the software tool technically sound?

Yes

Are sufficient details of the code, methods and analysis (if applicable) provided to allow replication of the software development and its use by others?

Yes

Is sufficient information provided to allow interpretation of the expected output datasets and any results generated using the tool?

Yes

Reviewer Expertise:

cell biology, genetic mutation

I confirm that I have read this submission and believe that I have an appropriate level of expertise to confirm that it is of an acceptable scientific standard, however I have significant reservations, as outlined above.

# Response

**Published:** 2022-07-14

**Authors:** Claudia Pommerenke

We thank the reviewer for their valuable comments. Please find our responses here:

**Major **

- *"In the RNA-seq heat map analysis, the clustering results performed based on the similarity of the expression profile of each gene are displayed, but the clustering result based on the similarity of the expression profile of each cell line should also be displayed."*
   
  - Thank you for your note on the heat map display. Clustering by cell line has been offered as option in the past, albeit by cell line solely without gene clustering. We now enabled clustering by cell line line and gene simultaneously (option “both”).

**Minor**

- *"The legend in Fig. 4B is not appropriate. There are no cell lines highlighted in yellow, while there is no indication of the color of cells highlighted in red."*
   
  - Many thanks to the reviewer for the hint. We have now displayed the colors correctly in Fig. 4B and described the cell lines highlighted in red as genetically unbiased.
- *"Although the data in Fig. 4B shows only 9 loci data as an example of LOH, it is considered that 17 loci data is collected in this database, so please describe the verification using all the data."*
   
  - Figure 4B has been redone so that all STR locations are now captured and the matches across STR 17 are completely shown.
- *"Also, why not explain the example of MSI-H using MOLT-4 etc.?"*
   
  - An explanation of the MSI phenomenon based on MOLT-4 would unfortunately go beyond the scope of this paper. For a satisfactory explanation, the failure of DNA MMR and the origin of DNA replication errors would have to be presented, so that the scope in this manuscript would be exceeded. In addition, it would need to be discussed much more deeply that the rule established by Amanda Capes-Davies for relatedness of cell lines when there is at least 80% similarity of the STR profile does not apply to MSI cell lines (Capes‐Davis, Amanda, et al. "Match criteria for human cell line authentication: where do we draw the line?" International journal of cancer 132.11 (2013): 2510-2519). Finally, MSI lines need additional tests as described in the STR standard of 2021 (ANSI/ATCC ASN-0002-2021 (2021) Authentication Of human cell lines: standardization of Short Tandem Repeat (STR) profiling. ANSI eStandards Store).

# Peer review: Reviewer response for version 1

**DOI:** 10.5256/f1000research.122868.r135181

**Published:** 2022-05-24

**Authors:** Beate Rinner

**Affiliations:** 1 Department for Biomedical Research, Medical University of Graz, Graz, Austria; 2 BioTechMed-Graz, 8010 Graz, Austria

In the submitted article of Koblitz et al., DSMZCellDive, a comprehensive data source for human and animal cells, will be presented, one aim is to provide high-throughput information through a web portal that aggregates data and references that are otherwise spread across multiple sources. 

 A variety of data sources are generated, such as RNA-seq transcriptome data and STR (Short Tandem Repeats) profiles, which are essential for working with cell lines. 

 The new tool is explained and described in detail, e.g. how to access the database via browsing, searching or visualization. The database can be extended with meta- and high-throughput data that will be generated in the future to finally allows a comprehensive summary and detailed characterizations of a cell line.

 To illustrate the power of the tool, analysis of B-cell differentiation markers, homeo-oncogenes, homeo-oncogene expression, and measurement of genomic loss of heterozygotes through an expanded STR panel of 17 loci were described. 

 DSMZCellDive offers enormous added value to the scientific community by supporting the selection of suitable model cell lines and thus ensuring the reliability and reproducibility of experiments. 

 Optimal in vitro conditions also save resources in the long term. The comprehensive data acquisition and provision of the tool leads to a massive upgrading of in vitro models, which enables the reduction of animal experiments and thus sustainably implements the 3R principles in research.

Are the conclusions about the tool and its performance adequately supported by the findings presented in the article?

Yes

Is the rationale for developing the new software tool clearly explained?

Yes

Is the description of the software tool technically sound?

Yes

Are sufficient details of the code, methods and analysis (if applicable) provided to allow replication of the software development and its use by others?

Yes

Is sufficient information provided to allow interpretation of the expected output datasets and any results generated using the tool?

Yes

Reviewer Expertise:

Cell line establishment, Cell line characterization, cell line quality check

I confirm that I have read this submission and believe that I have an appropriate level of expertise to confirm that it is of an acceptable scientific standard.

# Response

**Published:** 2022-07-14

**Authors:** Claudia Pommerenke

We thank the reviewer for the evaluation of our work and appreciate the positive comments.
